# Temperature Dependence of Conformational Relaxation of Poly(ethylene oxide) Melts

**DOI:** 10.3390/polym13224049

**Published:** 2021-11-22

**Authors:** Hye Sol Kim, Taejin Kwon, Chung Bin Park, Bong June Sung

**Affiliations:** Department of Chemistry, Sogang University, Seoul 04107, Korea; ihs545742@gmail.com (H.S.K.); taejin1112@sogang.ac.kr (T.K.); jungbin919191@gmail.com (C.B.P.)

**Keywords:** polymer melts, PEO, temperature dependence, Rouse model, conformation

## Abstract

The time-temperature superposition (TTS) principle, employed extensively for the analysis of polymer dynamics, is based on the assumption that the different normal modes of polymer chains would experience identical temperature dependence. We aim to test the critical assumption for TTS principle by investigating poly(ethylene oxide) (PEO) melts, which have been considered excellent solid polyelectrolytes. In this work, we perform all-atom molecular dynamics simulations up to 300 ns at a range of temperatures for PEO melts. We find from our simulations that the conformations of strands of PEO chains in melts show ideal chain statistics when the strand consists of at least 10 monomers. At the temperature range of T= 400 to 300 K, the mean-square displacements ($\langle \Delta r^{2}\left(t\right)\rangle$) of the centers of mass of chains enter the Fickian regime, i.e., $\langle \Delta r^{2}\left(t\right)\rangle ∼t^{1}$. On the other hand, $\langle \Delta r^{2}\left(t\right)\rangle$ of the monomers of the chains scales as $\langle \Delta r^{2}\left(t\right)\rangle ∼t^{1/2}$ at intermediate time scales as expected for the Rouse model. We investigate various relaxation modes of the polymer chains and their relaxation times ($\tau_{n}$), by calculating for each strand of *n* monomers. Interestingly, different normal modes of the PEO chains experience identical temperature dependence, thus indicating that the TTS principle would hold for the given temperature range.

## 1. Introduction

The time-temperature superposition (TTS) principle has been employed extensively when one tried to analyze viscoelastic and mechanical properties of polymeric systems [1,2,3,4,5]. TTS principle is very useful because one may superimpose linear viscoelastic data obtained at different temperatures and construct a master curve. The master curve provides the information on the mechanical properties at a wide temporal scale, which would be inaccessible without the TTS principle. It has been reported for decades, however, that the TTS principle often broke down in various polymeric systems near the glass transition [6,7,8,9,10,11,12,13,14,15,16]. The TTS principle is based on the underlying assumption that the various relaxation modes of a polymer chain would experience identical friction and hence the relaxational dynamics of those modes would couple to each other with the same temperature dependence. It should be, therefore, of academic interest to test the assumption for the TTS principle. In this study, we investigate the temperature dependence of various relaxation modes of poly(ethylene oxide) (PEO) chains by performing extensive all-atom molecular dynamics simulations for up to 300 ns.

The Rouse model is a successful model to describe the polymer dynamics and the conformational relaxations for unentangled polymer chains in melts. In the Rouse model, the friction coefficient ($\zeta_{R}$) that a polymer chain of degree of polymerization (*N*) experiences is proportional to *N*, i.e., $\zeta_{R}∼N^{1}$. The translational relaxation time called the Rouse time ($\tau_{R}$) is the time taken for the chain diffuses by its own size (i.e., $\tau_{R}\approx R_{g}^{2}/D$), where $R_{g}$ and $D∼1/\zeta_{R}$ are the radius of gyration and the diffusion coefficient of the chains, respectively. In case the chain conformations follow the ideal chain statistics ($R_{g}^{2}∼N$) as expected for polymer melts, $\tau_{R}$ scales as $\tau_{R}∼N^{2}$. The Rouse model also suggests that the polymer chain conformations relax such that the mean-square displacement ($\langle \Delta r^{2}\left(t\right)\rangle$) of monomers scales as $\langle \Delta r^{2}\left(t\right)\rangle ∼t^{1/2}$ for $t\leq \tau_{R}$. We confirm in our simulations that PEO chains in melts follow the Rouse model faithfully at a temperature range of T= 400 to 300 K [17].

The assumption that all the relaxation modes would have the same temperature dependence is implemented in various models including the Rouse and the Zimm models. In those models, the relaxation time (τ) of a certain relaxation mode is considered to be the product of the temperature-independent factor and the relaxation time ($\tau_{0}$) of monomers, which results in the same temperature dependence of various relaxation modes. τ is determined by the ratio of the friction coefficient (ζ) and *T*, i.e., τ∼ζ/T. The temperature dependence of ζ determines, therefore, the temperature dependence of τ. It has been well known that the friction coefficient (ζ) would increase roughly by an order of magnitude if *T* were to decrease by 3 K near the glass transition. On the other hand, far above the glass transition temperature ($T_{g}$), ζ increases roughly by a factor of 10 when *T* decreases by about 25 K [18,19]. In this study, we investigate the temperature dependence of various modes at temperatures above $T_{g}+25K$ and estimate the relaxation times (τ’s) at four orders of magnitude. We show that the assumption of the identical temperature dependence of relaxation times holds properly.

Molecular simulations can provide detailed information on the segmental and chain relaxation processes at a molecular level. Bormuth et al. performed all-atom molecular dynamics simulations for poly(propylene oxide) chains that consist of 2 to 100 monomers [20]. They found that α relaxations of chains of different length showed identical temperature dependence at sufficiently low temperatures such that TTS principle should hold. Tsalikis et al. employed the united-atom model for chains and performed extensive molecular dynamics simulations for both ring and linear PEO chains [21,22]. They compared their results with experiments and showed that molecular simulations could provide accurate information on the density, the conformation, and the segmental dynamics. They also showed that the chain dynamics at T=413 K, which is well above the $T_{g}$, followed the Rouse model faithfully. Motivated by the work by Tsalikis et al., we also consider PEO melts, but we focus on the temperature dependence of various relaxation modes of PEO chains and show whether those modes exhibit the same temperature dependence.

PEO melts are used in various products such as cosmetic, pharmaceuticals, and especially the next generation solid state electrolytes [23,24,25,26,27,28]. Because of the extensive applicability of PEO, there have been many simulation studies [29,30,31,32,33,34,35,36,37,38,39,40,41,42,43,44,45], which enables us to perform molecular dynamics simulations rather systematically. PEO melts have been considered as a strong candidate for solid polyelectrolytes. It has been proposed that a lithium ion in the solid PEO polyelectrolyte would migrate via three different mechanisms [46]: (1) the lithium ion diffuses along the PEO chain at short times, (2) the transport of lithium ion is accompanied by the conformational change of the PEO chain (that the lithium ion is attached to) at intermediate time scales, and (3) the lithium ion hops between two PEO chains at long time scales. This indicates that the conformational relaxation and the transport of PEO chains should be critical to understanding the conductivity of lithium ions in solid PEO polyelectrolytes. Therefore, it should be of importance to investigate the PEO conformational relaxation and its temperature dependence.

The rest of the paper is organized as follows: in Section 2, we discuss the simulation model and methods in details. Simulation results are presented and discussed in Section 3. Section 4 contains the summary and conclusions.

## 2. Materials and Methods

We perform atomistic molecular dynamics (MD) simulations for the melts of poly(ethylene oxide) (PEO) by employing LAMMPS (large-scale atomic/molecular massively parallel simulator) software [47]. Luo and Jiang carried out MD simulations for PEO melts of the degree of polymerization (*N*) of 50 and investigated the glass transition, Flory–Huggins parameters, and other structural parameters [48]. They found that their results for PEO melts of *N* = 50 were consistent with available experiments. Similarly, we prepare initial configurations of 20 PEO chains of *N* = 50 with periodic boundary conditions in all directions. The repeating unit of a PEO chain is $-[CH_{2}-O-CH_{2}]-$. The head and tail groups are $[CH_{3}-O-CH_{2}]-$ and $-[CH_{2}-O-CH_{3}]$, respectively. Our simulation system consists of 7040 atoms in total.

We describe PEO chains by using the OPLS-AA force field [49]. A spherical cutoff of 10 Å is imposed to the Lennard–Jones interactions. The long-range electrostatic contributions are calculated by using the Ewald summation. We ensure that the whole systems are neutral in the charges, and we scale down the charges by a factor of 0.8. Due to the scaling down of the charge, the density of PEO melts is about 1.071 g/cm${}^{3}$ at 300 K, which is consistent with the experimental data of 1.112 g/cm${}^{3}$ [50] and 1.07−1.27 g/cm${}^{3}$ [48].

We propagate the systems under isothermal-isobaric conditions with the velocity Verlet integrators [51]. The integration time step is 1 fs. A Nose–Hoover thermostat and barostat [52] are used at 1 atm and a given temperature. We equilibrate our systems for at least 20 ns at each temperature and check that the potential energy converges. Then, we propagate our systems up to 300 ns at each temperature. The ensemble averages of properties are obtained over up to five different sets of simulations at each temperature. Note, however, that even though we perform quite extensive MD simulations up to 300 ns, the polymer dynamics near the glass transition temperature is still slow such that the center of mass of each polymer chain does not diffuse much yet. For example, at *T* = 275 K, the center of mass of each polymer chain diffuses only by about its monomer size during 300 ns while the smaller segments of the chain diffuse more significantly. We find, however, that the diffusion of the center of mass of each chain becomes Fickian at T≥300 K. Because the slowest translational mode of chains, the diffusion of the centers of mass relaxes within 300 ns, and we expect that the polymer chain would keep undergoing normal diffusion beyond t = 300 ns.

In order to identify the glass transition temperature of PEO melts, we cool down PEO melts in a stepwise fashion from 400 to 160 K by a decrement of 20 K [53]. The cooling rate in our simulations is 4 × 10${}^{9}$ K/s, which is much higher than in experiments but is still close to previous simulations [40]. As shall be discussed below, $T_{g}$ = 249 K from our simulations is consistent with both previous experiments and simulation results [54,55].

We quantify the translational chain dynamics on a strand-length basis via the self-part of the intermediate scattering function, $F_{s}\left(q,t\right)=\langle \exp\left[-iq\cdot \left(r_{j}\left(t\right)-r_{j}\left(0\right)\right)\right]\rangle$. Here, q is the wavevector and $r_{j}\left(t\right)$ is the position vector of the center of mass of a strand *j* at time *t*. 〈⋯〉 represents an ensemble averaging. In order to calculate $F_{s}\left(q,t\right)$ for a strand of length n=N/p, we first divide each PEO chain into *p* strands of length *n*. Then, we locate the center of mass ($r_{j}\left(t\right)$) for each of those *n*-strands as a function of time. Note that the position vector of an oxygen atom of each monomer is taken as the position vector of a single monomer in this study. In case n=1, the strand corresponds to a segment, whereas n=N corresponds to a whole chain. We consider non-overlapping strands with *n* = 1, 2, 5, 10, 25, and 50 (*p* = 50, 25, 10, 5, 2, and 1, respectively). Once we calculate $F_{s}\left(q,t\right)$ from our trajectories, we fit the simulation results to a Kohlrausch–Williams–Watts (KWW) stretched exponential function, $F_{s}\left(q=2.244,t\right)=\exp\left[-{\left(\frac{t}{\tau_{\mathrm{KWW}}}\right)}^{\beta }\right]$. Here, $\tau_{KWW}$ and β are fitting parameters. *q* = 2.244 represents the length scale that corresponds to the first peak of the radial distribution functions of oxygen atoms. We, then, define a relaxation time ($\tau_{n}$) for any strand of length *n* by employing the equation of $F_{s}\left(q=2.244,t=\tau_{n}\right)=0.2$. Since all the simulation results for $F_{s}\left(q=2.244,t=\tau_{n}\right)$ decay well to 0 during our simulation times and the mean-square displacement of the centers of mass of chains diffuse beyond their own sizes at T≥300 K, we believe that 300 ns would be long enough to investigate the relaxations of various modes.

We calculate the mean-squared displacement (MSD) of strands of length n as follows:

(1) $$\langle \Delta r^{2}\left(t\right)\rangle =\langle {\left(r_{i}\left(t\right)-r_{i}\left(0\right)\right)}^{2}\rangle .$$

Here, $r_{i}$ denotes the position vector of the center of mass of a strand *i* at time *t*. We also investigate the self-part of the van Hove correlation function ($G_{s}\left(r,t\right)=\langle \delta (r-|r_{i}\left(t\right)-r_{i}\left(0\right)|)\rangle )$ of each strand. If PEO chains were to follow the conventional Fickian diffusion, $G_{s}\left(r,t\right)$ is expected to be Gaussian [56,57,58]. In order to estimate how much the diffusion of strands deviates from being Gaussian, we calculate the non-Gaussian parameter $\left(\alpha_{2}\left(t\right)\right)$ of strands of PEO chains as follows;

(2) $$\alpha_{2}\left(t\right)=\frac{3}{5}\frac{\langle \Delta r^{4}\left(t\right)\rangle }{{\langle \Delta r^{2}\left(t\right)\rangle }^{2}}-1.$$

Δr(t) is the displacement vector of a strand during time *t*. If a strand were to perform Gaussian diffusion, $\alpha_{2}\left(t\right)=0$.

We also monitor the rotational dynamics of a strand by calculating the rotational autocorrelation function, U(t) as follows [59]:

(3) $$U\left(t\right)=\langle \frac{r^{l}\left(t\right)r^{l}\left(0\right)}{r^{l}\left(t\right)r^{l}\left(0\right)}\rangle .$$

$r^{l}\left(t\right)$ stands for the end-to-end vector of each strand. For example, in the case of the rotational dynamics of a whole chain of n=50, $r^{l}\left(t\right)$ is the end-to-end vector of a chain, i.e., $r^{l}\left(t\right)$ = $r_{1}$ − $r_{50}$. $r_{1}$ and $r_{50}$ are the position vectors of the oxygen atoms of the first and the last monomers, respectively, at time *t*. For the rotational dynamics of a segment, $r^{l}\left(t\right)$ is a vector that connects two neighbor monomers, i.e., $r^{l}\left(t\right)$ = $r_{i}$ − $r_{i+1}$.

## 3. Results and Discussion

### 3.1. The Rouse Dynamics of PEO Melts

**Figure 1:**
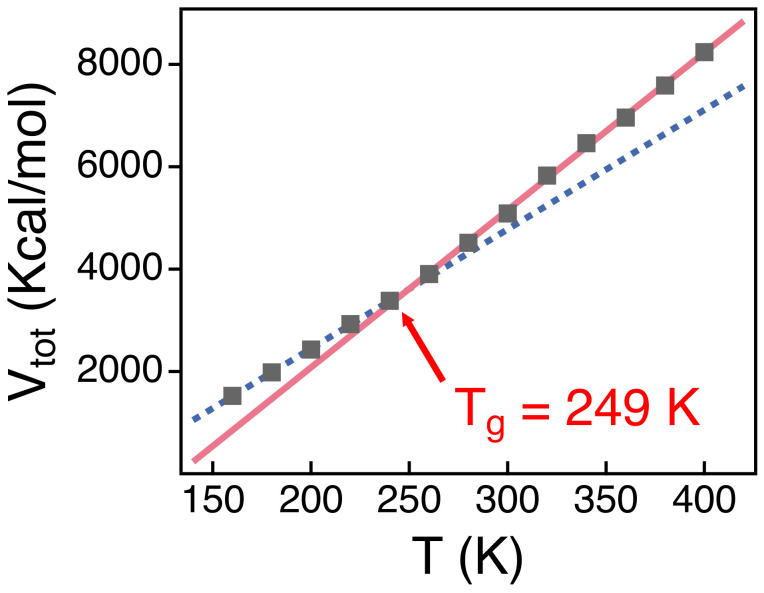
Simulation results for the total potential energy ($V_{tot}$) of the simulation systems as a function of temperature *T*. A solid line and a dashed line are linear fits to $V_{tot}$’s for the temperature ranges of 300–400 K and 160–230 K, respectively. The intersection of two fitting lines is estimated to be $T_{g}$. The statistical errors are smaller than the markers.

The dynamics of polymer chains in melts become spatially heterogeneous as temperature decreases toward the glass transition temperature ($T_{g}$). $T_{g}$ of PEO melts of a high molecular weight ranged between 158 and 233 K [54,55]. A previous simulation study for PEO melts of *N* = 50 also reported $T_{g}\approx$ 251 K [40]. In order to verify the simulation model employed in this study, we investigate $T_{g}$ from our simulations. We calculate the total potential energy ($V_{tot}$) of our simulation system as a function of temperature (*T*) (Figure 1). The slope of $V_{tot}$ changes at T= 249 K as indicated by two guide lines in the figure. This suggests that $T_{g}=$ 249 K for our simulation system, which is consistent with previous studies [31,40]. In this study, we focus the conformation and the dynamics of polymer chains well above $T_{g}$, where we may equilibrate our simulation systems and investigate the temperature-dependence of conformational relaxations readily.

**Figure 2:**
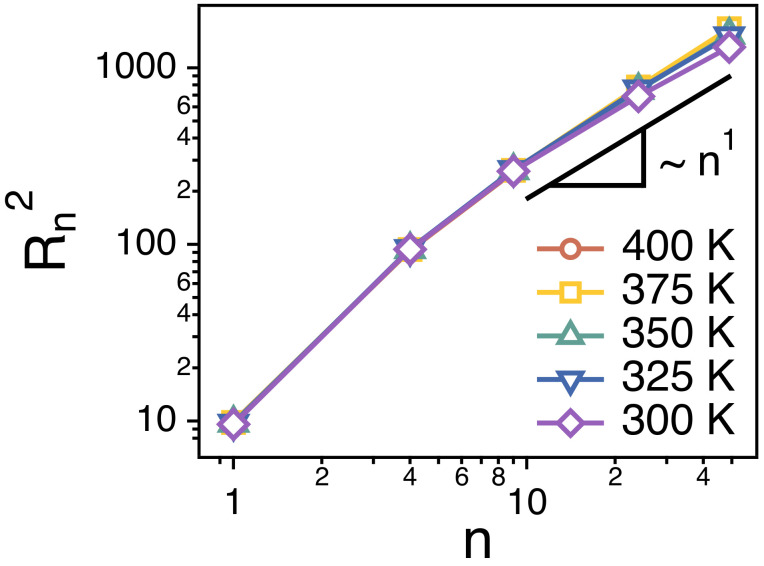
Simulation results for the end-to-end distance ($R_{n}$) of strands of size *n* at different temperatures. A solid line is a guide with an exponent of 1.

In a monodisperse polymer melt, chain conformations are expected to have ideal statistics. The number ($g_{T}$) of monomers in a thermal blob (a thermal length scale below which the excluded volume interactions are small compared to $k_{B}T$ such that the chain conformations are ideal) is $N^{2}$. Since the whole chain consists of only *N* monomers much smaller than $g_{T}=N^{2}$, chains are supposed to behave like ideal chains. In this study, instead of changing the values of *N* of the chains and estimating the size of the chains, we investigate the end-to-end distance ($R_{n}$) of each strand of different length *n*. We find that, for sufficiently large strands of n≥10, $R_{n}^{2}∼n^{1}$ such that the conformations of long strands are ideal (Figure 2).

**Figure 3:**
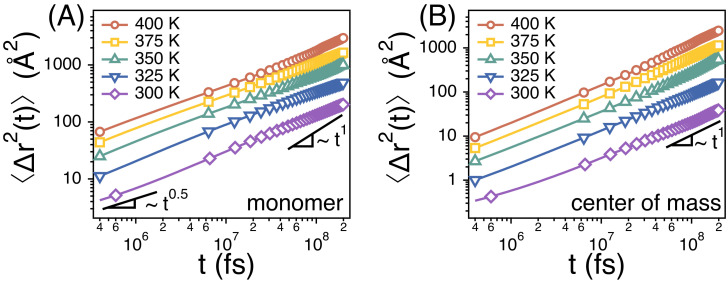
Simulation results for the mean-square displacements ($\langle \Delta r^{2}\left(t\right)\rangle$) of (**A**) monomers and (**B**) the centers of mass of PEO chains at different temperatures. Solid lines are guides with exponents of 1 and 1/2. The statistical errors are smaller than the markers.

The translational diffusion of a whole chain is Fickian in our simulation times of 300 ns at temperatures from 400 K to 300 K (Figure 3B), i.e., $\langle \Delta r^{2}\left(t\right)\rangle$ of the center of mass of chains is linear with time *t*. We also perform simulations at lower temperatures around 275 K, but the systems do not reach equilibrium within 300 ns at lower temperatures. Unless otherwise noted, we focus on the simulation results above *T* = 300 K. At *T* = 400 K, $\langle \Delta r^{2}\left(t\right)\rangle$ increases beyond $R_{g}^{2}$ at long times, thus indicating that the chains diffuse by more than their own size. Here, $R_{g}$ is the average radius of gyration of whole chains, and $R_{g}^{2}\approx$ 257.3 at 400 K. At *T* = 300 K, however, $\langle \Delta r^{2}\left(t\right)\rangle \approx$ 50 at *t* = 300 ns such that the chains may not diffuse much beyond its own size.

Rouse model predicts that, at an intermediate time scale between $\tau_{0}$ and $\tau_{R}$, $\langle \Delta r^{2}\left(t\right)\rangle$ of monomers of chains scales as $\langle \Delta r^{2}\left(t\right)\rangle ∼t^{1/2}$. $\tau_{0}$ denotes the monomer relaxation time and corresponds to the time taken for a monomer to diffuse by its own size. On the other hand, $\tau_{R}$ corresponds to the Rouse time at which the chain diffuses by its own size. We also find from our simulations that $\langle \Delta r^{2}\left(t\right)\rangle$ of monomers scales as $\langle \Delta r^{2}\left(t\right)\rangle ∼t^{1/2}$ (Figure 3A), thus indicating that the chain dynamics follows the Rouse model faithfully in our study.

**Figure 4:**
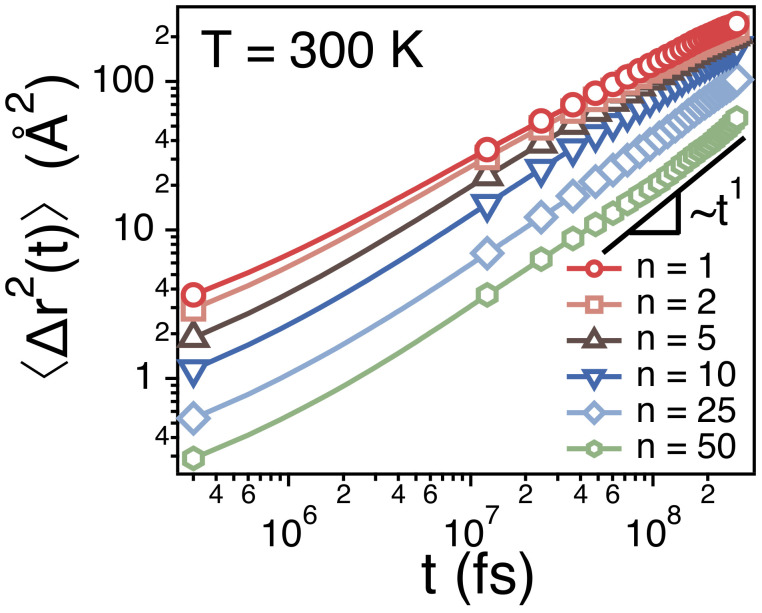
Simulation results for the mean-square displacements ($\langle \Delta r^{2}\left(t\right)\rangle$) of the centers of mass of strands of size *n* at *T* = 300 K. The statistical errors are smaller than the markers.

Figure 4 depicts $\langle \Delta r^{2}\left(t\right)\rangle$’s of centers of mass of different strands. Note that the strands of n=1 and n=50 correspond to the monomer and the whole chain, respectively. As discussed earlier, $\langle \Delta r^{2}\left(t\right)\rangle ∼t^{1/2}$ for a monomer (the strand of n=1) and $\langle \Delta r^{2}\left(t\right)\rangle ∼t^{1}$ for a whole chain (the strand of n=50). When the size (*n*) of strands is small, the dynamics of the strands is subdiffusive such that $\langle \Delta r^{2}\left(t\right)\rangle ∼t^{\alpha }$ and 0.5≤α<1. As *n* increases, the time exponent for the subdiffusion also increases gradually to 1.

**Figure 5:**
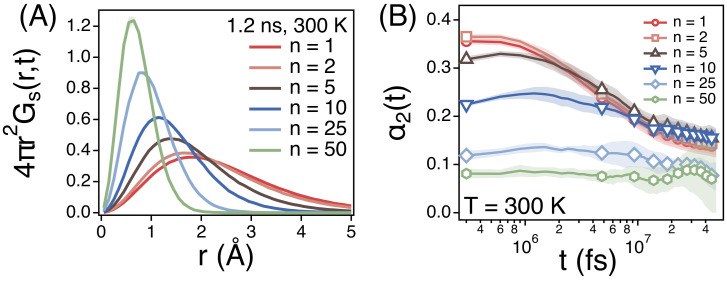
Simulation results for (**A**) the self-part of van Hove correlation functions ($G_{s}(r,t$ = 1.2 ns)) and (**B**) the non-Gaussian parameters ($\alpha_{2}\left(t\right)$) of the centers of mass of strands of size *n* at *T* = 300 K. Each shade represents a statistical error.

We also investigate the self-part of van Hove correlation function ($G_{s}\left(r,t\right)$) of the centers of mass of different strands at *t* = 1.2 ns (Figure 5A) at 300 K. $G_{s}\left(r,t=1.2\;ns\right)$ indicates the distribution function of the distance that strands diffuse during 1.2 ns. As expected from $\langle \Delta r^{2}\left(t\right)\rangle$ of strands, smaller strands diffuse much longer distance and $G_{s}\left(r,t\right)$’s of smaller strands are distributed more broadly. However, the diffusion of smaller strands is more non-Gaussian. Figure 5B depicts the non-Gaussian parameter ($\alpha_{2}\left(t\right)$) of strands. For large strands of *n* = 25 and 50, $\alpha_{2}\left(t\right)$ is relatively small around $\alpha_{2}\left(t\right)\approx 0.1$ at all time scales. This is because the diffusion of the center of mass of chains enters the Fickian regime such that the center of mass of chains undergoes the normal diffusion. For smaller strands, $\alpha_{2}\left(t\right)$ is relatively large especially at early times. This is because the diffusion of small strands is subdiffusive with the time exponent α<<1.

**Figure 6:**
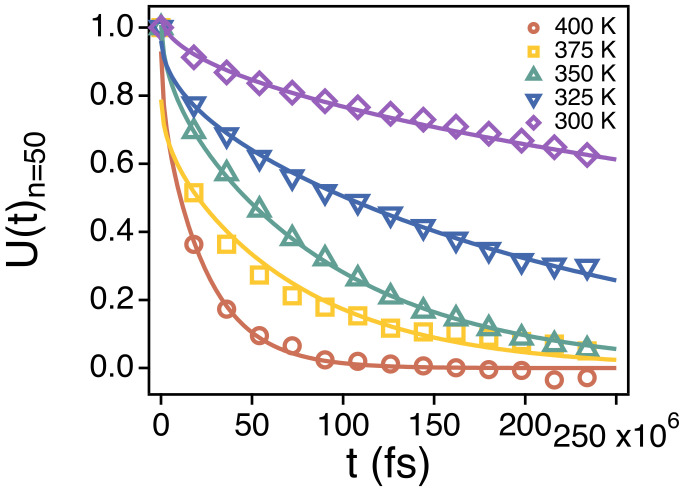
The end-to-end vector time correlation function U(t) of the whole chain (*n* = 50) at different temperatures. The solid lines are fits to Equation (4) for the observed temperatures. The statistical errors are smaller than the markers.

According to the Rouse model, the time correlation function (U(t)) of the end-to-end vector is expected to be expressed as the sum of relaxations of various modes as follows:

(4) $$U\left(t\right)=U_{N}\sum_{odd\;p}\frac{1}{p^{2}}\exp\left(-\frac{p^{2}}{2\tau_{R}}t\right),$$

where $U_{N}$ is the normalized constant and *p* ranges from 1 to 50. Note that, as shown in Equation (3), U(t) is normalized in our study. Our simulation results for U(t) for the end-to-end vector of chains are in good agreement with the above equation at all temperatures (Figure 6). In Figure 6, the symbols and the lines are the simulation results and fits based on the Equation (4), respectively. This indicates that even the orientational relaxation of the PEO chains at *T* from 300 to 400 K in this study follow the Rouse model quite faithfully.

**Figure 7:**
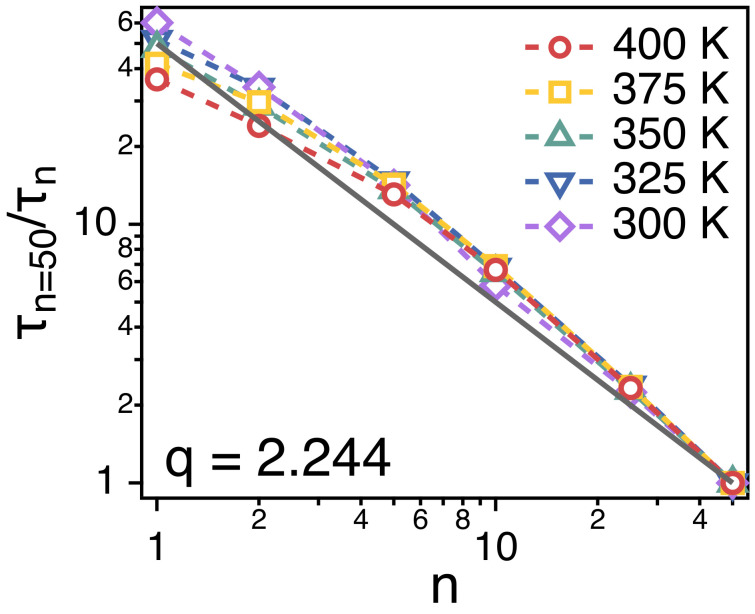
Simulation results for the relative relaxation times ($\tau_{n}$ of spatiotemporal correlations of strands of size *n*. The solid line is a guide line of $\tau_{n=50}/\tau_{n}∼n^{-1}$.

Figure 7 depicts the relaxation time $\tau_{n}$ of each strand. $\tau_{n}$ is obtained by fitting the simulation results for $F_{s}\left(q=2.244,t\right)$ of each strand to the stretched exponential function, $F_{s}\left(q=2.244,t\right)=\exp\left[-{\left(\frac{t}{\tau_{\mathrm{KWW}}}\right)}^{\beta }\right]$. $\tau_{n}$ is expected to be proportional to the ratio of the friction coefficient ($\zeta_{n}$) and temperature (*T*), i.e., $\tau_{n}∼\zeta_{n}/T$. In Figure 7, we divide the relaxation time ($\tau_{n=50}$) of a whole chain by $\tau_{n}$ of strands of *n*. For all of the strand length, $\tau_{n=50}/\tau_{n}∼n^{-1}$. This indicates that the friction ($\zeta_{n}$) that a strand of *n* monomers experience is proportional to *n*, i.e., $\zeta_{n}∼n^{1}$, which corroborates the main assumption of Rouse model.

### 3.2. Temperature Dependence of Conformational Relaxation

**Figure 8:**
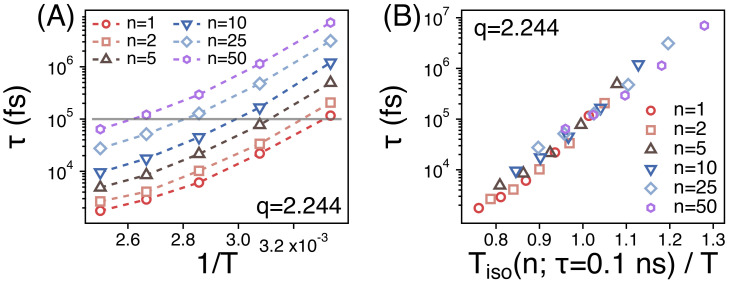
(**A**) The relaxation times ($\tau_{n}$) of spatiotemporal correlations of strands of size *n* as functions of 1/T; (**B**) $\tau_{n}$ as a function of the rescaled temperature. $T(n;\tau_{n}=0.1$ ns) is the temperature at which $\tau_{n}=0.1$ ns.

The spatiotemporal correlations of PEO melts relax readily in our simulations at *T* = 300 to 400 K. $F_{s}\left(q=2.244,t\right)$’s for strands of different size manage to decay below 0.2 within simulation times of 300 ns. The simulation results for $F_{s}\left(q,t\right)$ in our simulations are consistent with previous quasielastic neutron scattering experiments [29]. The relaxation time ($\tau_{n}$) is obtained as discussed in the above section. Figure 8A depicts the relaxation times ($\tau_{n}$) of different strands as a function of temperature (1/T). As shown in Figure 4, the segmental dynamics is much faster than the whole chain dynamics. As temperature decreases from 400 to 300 K, $\tau_{n}$ covers about two orders of magnitude of time scales. For example, $\tau_{n}$ increases from 0.06 to 7 ns for the strands of n=50.

In order to compare the temperature dependence of $\tau_{n}$ of different strands, we replot the Figure 8A by rescaling the abscissa. We introduce the temperature ($T_{iso}(n;\tau =0.1$ ns)) at which $\tau_{n}\approx 0.1$ ns. We rescale the temperature *T* by using $T_{iso}\left(n;\tau =0.1\;\mathrm{ns}\right)$ as in Figure 8B. Then, the values of $\tau_{n}$ of different strands manage to overlap well with one another within the simulation temperature range. This suggests that the relaxations of the spatiotemporal correlations of different strands should exhibit the same temperature dependence.

**Figure 9:**
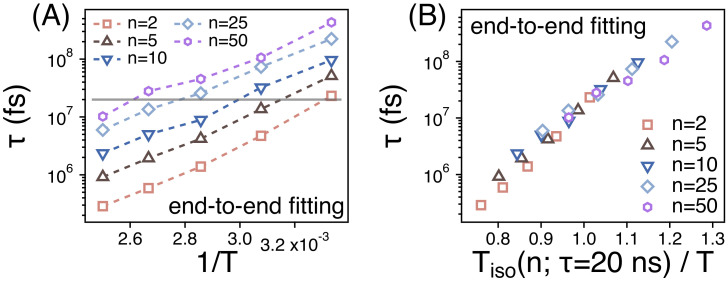
(**A**) The relaxation times ($\tau_{ete}$) of the orientational relaxation of strands of size *n* as functions of 1/T; (**B**) $\tau_{n}$ as a function of the rescaled temperature. $T(n;\tau_{ete}=20$ ns) is the temperature at which $\tau_{ete}=20$ ns.

We also investigate the relaxation of the orientational time correlation function (U(t)) of the end-to-end vector of different strands by estimating its relaxation time $\tau_{ete}$. $\tau_{ete}$ is also obtained by fitting the simulation results for U(t) to $U\left(t\right)=\exp[-{\left(\frac{t}{\tau_{ete}}\right)}^{\beta }]$. As shown in Figure 9A, for a given temperature and *n*, $\tau_{ete}$ is much larger than $\tau_{n}$ indicating that the orientational relaxation of a strand takes much a longer time than the relaxation of the spatiotemporal correlation. Just like $\tau_{n}$, however, $\tau_{ete}$ also covers about two orders of magnitude of time scales in our simulation temperatures. When we rescale the abscissa by introducing the temperature $T_{iso}(n;\tau_{ete}=20$ ns), $\tau_{ete}$’s of different strands overlap well with one another within the temperature range. This also indicates that the temperature dependence of the orientational relaxation of strands is identical regardless of *n*.

## 4. Conclusions

We investigate the dynamics and the temperature dependence of conformational relaxations in PEO melts. We perform extensive atomistic MD simulations for PEO melts at various temperatures up to 300 ns by employing the OPLS all-atom force field. We also investigate the potential energy of PEO melts by cooling the system and find that the glass transition temperature ($T_{g}$) is about 249 K, which is consistent with previous experimental and simulation studies.

The dynamics of strands of PEO melts follow the Rouse model faithfully at temperatures between 300 and 400 K. In our simulation times of 300 ns, the mean-square displacement ($\langle \Delta r^{2}\left(t\right)\rangle$) of the center of mass of whole chains enters a Fickian regime with $\langle \Delta r^{2}\left(t\right)\rangle ∼t^{1}$. In addition, $\langle \Delta r^{2}\left(t\right)\rangle$ of monomers scales as $\langle \Delta r^{2}\left(t\right)\rangle ∼t^{1/2}$ at the intermediate time scales before the Rouse time ($\tau_{R}$), which is expected for the Rouse model. We also find from simulation results for U(t) and relaxation times that the friction ($\zeta_{n}$) (that a strand of *n* monomers experience) is proportional to the number (*n*) of monomers in the strand, which is consistent with the Rouse model.

We investigate the relaxation of spatiotemporal correlation and conformations of strands of PEO chains at different temperatures. Between 300 and 400 K, the relaxation times change by about two orders of magnitude and depend strongly on the size (*n*) of strands. However, both $\tau_{n}$ and $\tau_{ete}$ of various strands exhibit identical temperature dependence. This corroborates the assumption for the time-temperature superposition principle that the relaxation of various conformational modes of a chain would exhibit the same temperature-dependence. In our future study, we plan to investigate the temperature dependence of conformational relaxations either near the glass transition temperature or with ions such as lithium ions mixed in melts.

## Data Availability

The data presented in this study are available on request from the corresponding author.
